# Herbal Medicine* Cordyceps sinensis* Improves Health-Related Quality of Life in Moderate-to-Severe Asthma

**DOI:** 10.1155/2016/6134593

**Published:** 2016-12-05

**Authors:** Ningqun Wang, Jie Li, Xiaobo Huang, Wenqiang Chen, Yujing Chen

**Affiliations:** ^1^Department of Traditional Chinese Medicine, Xuanwu Hospital, Capital Medical University, 45 Changchun Street, Beijing 100053, China; ^2^Department of Respiratory Medicine, Dongzhimen Hospital of Beijing University of Chinese Medicine, Beijing 100700, China

## Abstract

Moderate-to-severe asthma has a substantial impact on the health-related quality of life (HR-QOL) of the patients.* Cordyceps sinensis* is a traditional Chinese medicine that is evaluated clinically for the treatment of many diseases, such as chronic allograft nephropathy, diabetic kidney disease, and lung fibrosis. In order to investigate the effects of* Cordyceps sinensis* on patients with moderate-to-severe persistent asthma, 120 subjects were randomized to receive Corbin capsule containing* Cordyceps sinensis* for 3 months (treatment group, *n* = 60), whereas the control group (*n* = 60) did not receive treatment with Corbin capsule. Inhaled corticosteroid and as-needed *β*-agonists were used in the treatment of both groups. HR-QOL was measured with the Juniper's Asthma Quality of Life Questionnaire (AQLQ). The incidence of asthma exacerbation, pulmonary function testing, and serum measurements of inflammatory mediators were also evaluated. The results showed that the treatment group indicated a significant increase in AQLQ scores and lung function compared with the control group. The expression levels of the inflammation markers IgE, ICAM-1, IL-4, and MMP-9 in the serum were decreased and IgG increased in the treatment group compared with the control group. Therefore, the conclusion was reached that a formulation of* Cordyceps sinensis* improved the HR-QOL, asthma symptoms, lung function, and inflammatory profile of the patients with moderate-to-severe asthma. This trial is registered with ChiCTR-IPC-16008730.

## 1. Introduction

Asthma is a chronic disease of the upper respiratory tract that occurs as a result of bronchial hyperinflammation and airway hyperresponsiveness. A high percentage of patients suffer from uncontrolled asthma that is attributed to increased secretion of IgE, resulting from the recruitment of inflammatory cells such as Th2 cells and eosinophils to the airway tissue and the bronchoalveolar fluid [1–6]. These patients are at high risk of asthma-associated mortality and hospitalization and experience significant impairments in the quality of life [7]. The disease may have varying degrees of impact on the physical, psychological, and social wellbeing of people [7, 8]. Subjects with asthma are less likely to report excellent health, compared with people without the condition, whereas asthma ranks fourth, after cancer and kidney disease in the list of diseases that force people to request time off work, school, or study [9]. Moreover, patients with asthma often experience comorbidities, such as rhinitis, obesity, and cardiovascular disease [10]. Thus, the international guidelines for asthma therapies aim to achieve and maintain long-term control of the disease, in order to improve the quality of life of the patient by minimizing the symptoms and improving physical, psychological, and social function. Indeed, improvements in health-related QOL (HR-QOL) may be more reflective of treatment success, compared with physiological endpoints such as spirometric measures of lung function [11]. The Global Initiative for Asthma (GINA) guidelines recommend a stepwise approach to the treatment of asthma that is based on a combination of assessment, treatment, and monitoring strategies [12]. The initial treatment recommended consists of low dose-inhaled corticosteroids (ICS), whereas when symptoms persist a treatment strategy with a long-acting *β*2-agonist (LABA), such as formoterol or salmeterol, and/or a sustained dose of a leukotriene modifier is considered, before increasing the dose of ICS [12, 13]. Recently, the use of anti-IgE treatment with a recombinant humanized monoclonal antibody designated as Omalizumab is being explored as an alternative [13]. However, despite the treatment strategies indicated by international guidelines, at least 40% of patients have symptomatic or poorly controlled disease, whereas the extensive use of ICS as a first-line treatment results in severe side effects, such as mouth infections, cataracts, and osteoporosis [14–17]. As a result, the exploration of new therapeutic avenues with limited adverse effects for the treatment of chronic asthma is imperative. In addition, several randomized clinical trials have been conducted, in order to assess the effectiveness and safety of combination therapies in human populations suffering from asthma, as regards the quality of life and the occurrence of asthma-associated comorbidities [12, 13].

Many patients with chronic allergic conditions seek complementary and alternative medicine therapies including traditional Chinese medicines. This trend has begun to attract interest from mainstream healthcare providers and scientific investigators [18–20].* Cordyceps sinensis *is a natural herbal medicine derived from the sac fungi that has been popular in China for centuries for health preservation and the reduction of fatigue. Due to the rare occurrence of* Cordyceps sinensis*,* Cordyceps militaris*, a similar herb containing the active ingredient of* Cordyceps,* is cultivated extensively by fermentation technology [21].* Cordyceps militaris *has been investigated as an antitumor, antimetastatic, immunomodulatory, and antioxidant regimen [21], while it is traditionally used in Korea, China, and Japan for the ethnopharmacological treatment of glucose metabolism, hypocholesterolemia, hepatic disease, and diabetes [22–24]. In addition,* Cordyceps* has demonstrated anti-inflammatory effects in* in vitro* and/or* in vivo* models, such as the LPS-induced Raw 264.7 macrophage cells and the ovalbumin-induced Balb/c mice [4, 25]. It has been reported that* Cordyceps* attenuated airway hyperresponsiveness, mucous hypersecretion, and ovalbumin-specific IgE production in a murine model of asthma, although the exact mechanism of action remains undiscovered [4].

Evidence regarding the anti-inflammatory effect of* Cordyceps* in patients with asthma is limited. To our knowledge, only one clinical study has been conducted in children with asthma using a herbal formula that is designated as CUF-2 capsule [26]. The present clinical study was designed to investigate the potential of the Chinese herb* Cordyceps sinensis *to improve the HR-QOL in asthma patients and reduce the extent of inflammation that results from chronic asthma. The expression levels of the classical inflammatory biomarkers such as IgG, IgE, MMP9, IFN-*γ*, IL-4, and ICAM-1 [27–30] that play an important role in the pathogenesis of asthma were also assessed.

## 2. Methods

### 2.1. Patients

The randomized controlled trial was conducted at Xuanwu Hospital during the period of January 2014 to December 2015. Enrolled subjects were at least 18 years old and were diagnosed with moderate or severe asthma with evidence of fixed airflow obstruction following a trial of maximum bronchodilator therapy and a trial of oral corticosteroids of at least 3-week duration. Moderate-to-severe asthma with fixed airflow obstruction was defined by forced expiratory volume in 1 s (FEV 1) < 80% predicted and FEV 1/forced vital capacity < 80% predicted. All enrolled subjects were receiving long-term management by a respiratory physician. Exclusion criteria included smoking history > 15 years; respiratory tract infection in the previous 4 weeks; evidence of coexisting respiratory conditions such as cystic fibrosis and chronic obstructive pulmonary disease; comorbid conditions likely to reduce exercise capacity; any significant uncontrolled disease state other than asthma; pregnancy or lactation; patients receiving immunotherapy; inability to complete baseline quality of life assessment; hospitalization for asthma within 3 months; mechanical ventilation during an asthma exacerbation within 2 years. Recruitment was restricted to patients from one respiratory physician at one hospital to minimize variations in asthma management. The ethical approval for the study was obtained from the Human Research Ethics Committees of Xuanwu Hospital, Capital Medical University. All subjects were given written informed consent prior to participation. This trial is registered with ChiCTR-IPC-16008730.

### 2.2. Interventions

A total of 120 subjects were randomized to treatment (*n* = 60) and control groups (*n* = 60), using simple randomization. According to the guidelines on asthma management outlined by the British Thoracic Society and Chinese Medical Association, inhaled corticosteroid and long-acting *β*2-adrenergic agonist were used in the control group. The treatment group orally received* Cordyceps sinensis* (1.2 g, 3 times per day, Corbrin capsule, Hangzhou Huadong Pharmaceutical Co. Ltd.) in addition to inhaled corticosteroid and long-acting *β*2-adrenergic agonist as needed. The intervention period was 3 months.

### 2.3. Outcomes and Follow-Up

#### 2.3.1. Primary Outcome

HR-QOL was measured with the Juniper's Asthma Quality of Life Questionnaire (AQLQ) 1 day before, 1 day after, and 3 months after the intervention period. The spirometry measurements, asthma control conditions, and serum IgG, IgE, MMP9, IFN-*γ*, IL-4, and ICAM-1 levels were evaluated before and after the treatment period.

#### 2.3.2. Other Outcomes

*Questionnaire.* AQLQ [31] is a 32-item scale assessing symptoms, activity limitations, and emotional and environmental effects of asthma. Each question was answered by the patient on a 7-point scale, with a score of 1 representing the greatest impairment and a score of 7 representing no impairment (a higher AQLQ score therefore reflects a better quality of life). All items have equal weights and the mean score for each domain (activity limitations, emotions, symptoms, and exposure to environmental stimuli) was calculated for each patient. The average overall score for the AQLQ was also calculated.

*Lung Function Tests.* The pulmonary function test was performed using a spirometer (RESMED3VPAP), according to the standards outlined by the ATS/ERS Task Force [32]. Forced vital capacity (FVC), mean peak expiratory flea (PEF), and forced expiratory volume in one second in liters (FEV1) were recorded.

*Measurement of Cytokine Levels.* The blood samples were collected before and after the intervention period. The serum concentrations of IgG, IgE, MMP9, IFN-*γ*, IL-4, and ICAM-1 were determined using commercially quantitative enzyme-linked immunosorbent assay (ELISA) kits (ab195215, ab108650, ab100610, ab46025, ab174449, and ab100640 from ABCAM, China), according to the manufacturer's instructions. For all ELISA assays, coating with capture antibodies, blocking, incubation of samples, and reaction with the detecting antibodies were performed at 4°C and the reaction time was prolonged to 8 hours to enhance the assay sensitivity.

### 2.4. Sample Size

Sample size calculation was performed based on the percentage of patients with an improvement of 0.5 points on the AQLQ score and the assumption that *α* = 0.05 and 1 − *β* = 0.8 and a 5% loss to follow-up [33]. A sample size of 60 patients will be sufficient.

### 2.5. Statistical Analysis

A double entry system of the data was used in SPSS (version 10.0; SPSS Inc., Chicago, IL, USA). The continuous variables are expressed as the mean ± standard deviation or median ± quartile according to data distribution. The categorical variables are expressed as percentages. The postintervention changes and 3-month changes from baseline data within each group were compared using a univariate Student's *t*-test or nonparametric test. A *χ* ^2^ test was used to compare categorical data. Comparison of continuous variables between two groups was performed using the independent samples *t*-test or Mann–Whitney *U* test. Pearson's correlations and Spearman's correlations were used to assess relationships between variables. A *p* value of <0.05 was considered significant.

## 3. Results

### 3.1. Characteristics of Subjects at Baseline

**Figure 1:**
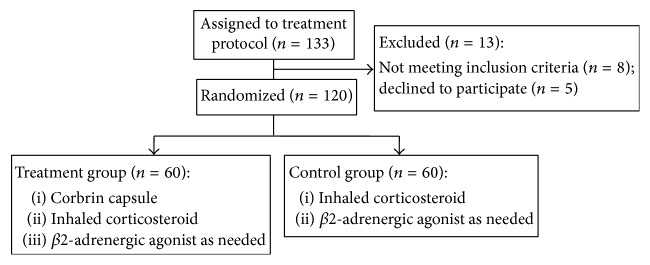
The control group cannot contain Corbrin capsule. Alternatively, a placebo capsule should have been used.

A total of 133 patients were assigned to the treatment protocol (Figure 1). The patients that did not meet the eligibility criteria (*n* = 8) were excluded from the study, whereas 5 patients declined to participate (Figure 1). A single randomization scheme was applied to 120 patients participating in the study (Figure 1). The subjects were divided into the treatment arm (*n* = 60) that was given Corbin capsule, containing 1.2 g of* Cordyceps sinensis*, orally 3 times per day, and the control arm (*n* = 60) that did not receive* Cordyceps sinensis* (Figure 1). All patients in the control and treatment groups were given standard therapy for asthma treatment that included corticosteroids and *β*2-adrenergic agonists (Figure 1). The intervention period was 3 months and the follow-up period was 3 months. No patients were withdrawn from the study. There were no significant differences in the demographic parameters or the baseline characteristics between the two treatment groups (*p* < 0.05, 0.01). The demographic and disease-related parameters of the patients who completed the study are presented in Table 1.

### 3.2. Comparison of AQLQ Score between Two Groups

No significant differences were observed between the two groups at the baseline time point. At postintervention assessment, the disease parameters that were evaluated such as “asthma symptom” and “concern for health” and overall AQLQ score were significantly increased in the treatment group compared with the baseline (*p* < 0.05). In the control group, the only parameter that was increased compared to the baseline over this period was “asthma symptoms” (*p* < 0.01). At the 3-month follow-up, all the parameters except “environment stimuli” were significantly increased in the treatment group compared with the baseline (*p* < 0.05) for “activity limitation,” “asthma symptom,” “emotional function,” and overall AQLQ. A significant increase was noted at the 3-month period in the treatment group in the parameters “activity limitation,” ”asthma symptom,” “emotional function” domains, and overall AQLQ score, compared with the control group (*p* < 0.05, 0.01) (Table 2).

### 3.3. Comparison of Lung Function and Serum Cytokines Level between Two Groups

The lung function, as indicated by the predicted FEV1, PEFR, and FEV1/FVC, was improved after 3 months of intervention with Corbrin in the treatment group compared to baseline data (*p* = 0.026, 0.041, and 0.000, resp.). The improvement of lung function in the treatment group was more significant than that of the control group (*p* < 0.05). In the treatment group, the IgG level increased after 3 months of intervention (*p* = 0.000). The IgE level decreased along with the levels of the markers ICAM-1, IL-4, and MMP-9 after 3 months of intervention (*p* = 0.017, 0.002, 0.000, and 0.000, resp.). The treatment group showed a marked decrease in inflammatory biomarkers IgE, ICAM-1, IL-4, and MMP-9 and an increase in IgG compared with the control group (*p* < 0.05, 0.001). The levels of IFN-*γ* were not significantly different between the two groups (Table 3).

### 3.4. Comparison of Asthma Exacerbation Indications between Two Groups

Treatment with Corbrin resulted in a significantly greater improvement in the symptom-free days compared with control group (*p* < 0.001). Similarly, the mean number of daytime onsets was significantly reduced with Corbrin treatment compared with the control (*p* < 0.05). Treatment with Corbrin resulted in significantly greater increases in the mean number of the days where patients did not use any rescue medication (*p* < 0.001). There were no significant differences between two groups regarding the change of nighttime onset, nocturnal awakenings, and average inhaled corticosteroids (Table 4).

### 3.5. Safety

Asthma exacerbations were reported in 5 patients (8.3%) treated with Corbrin and in 7 patients (11.6%) in the control group. The most common reason cited for the cause of asthma exacerbation was the infection of the respiratory tract. Adverse events were reported in 10% and 13% of patients treated with Corbrin and with standard therapy of asthma. The adverse events that were reported in the treatment group were headache, throat discomfort, and dry mouth (Table 5). No significant differences with respect to self-reported symptoms (adverse events) were observed between the participants assigned to the treatment group or control group (*p* > 0.05). All the symptoms disappeared spontaneously without intervention.

## 4. Discussion

The results of the present study demonstrate that concomitant treatment of the Corbrin capsule and standard therapy, including corticosteroids and *β*2-adrenergic agonists, can improve significantly the quality of life of patients suffering from asthma. The beneficial effects of Corbrin were noted as regards the parameters “activity limitation,” “asthma symptoms,” and “emotional function.” The effects were more profound at the 3-month follow-up period, compared with the postintervention period, and were possibly attributed to the improvement of lung function and asthma symptoms by Corbrin. In addition, Corbrin increased significantly the mean number of symptom-free days and the mean number of rescue-free days compared with the control group, while it decreased the mean number of daytime onsets.

Herbal medicines combined with routine pharmacotherapies were used in some previous studies in treatment of asthma. The integrated approach improved outcomes greater than pharmacotherapies alone. There were studies using herbal decoctions combined with routine pharmacotherapies that showed improving FEV1 and PEFR [34, 35]. Other studies showed fewer exacerbations and a significant reduction in use of rescue medication by treatment with herbal compound combined with routine pharmacotherapies [36–38]. Only one study reported quality of life outcome which showed herbal medicine plus pharmacotherapy was not different from pharmacotherapy alone [36]. Although the AQLQ outcome was included in some studies which investigated the effect of high dose-inhaled glucocorticoids or exercise training on quality of life in patients with asthma [39–41], it is seldom evaluated in studies with herbal medicines. To date, no study has investigated the effects of* Cordyceps sinensis *on asthma, especially on quality of life of asthma patients, although the protective effects of* Cordyceps *have been proved in many diseases such as diabetes, renal insufficiency, and contrast-induced nephropathy [42, 43] and a considerable number of studies explored the use of* Cordyceps* in exercise, performance, and endurance on human subjects [44, 45].

Our study showed positive effects of Corbrin on improving lung function and asthma severity. Furthermore, the quality of life was improved after 3 months of intervention with Corbrin. As a growing number of asthma patients are using or wish to use some form of complementary and alternative medicine (CAM) [46] and Corbrin is a widely accepted Chinese medicine in China, using Corbrin as complementary approach in treating asthma may result in better asthma control and better quality of life according to our results. The apparent lack of significant adverse effects also supports the use of Corbrin for the treatment of asthma. Further, other herbs have not shown similar definitive improvements in asthma quality of life suggesting that Corbrin may have uniquely effective features.

One of the major consequences of the pathogenesis of asthma is the infiltration of the inflammatory cells in the lung tissue [47]. The present study suggests a protective effect of* Cordyceps sinensis* against inflammation during asthma as measured by several serum biomarkers.* Cordyceps sinensis* may indirectly downregulate ICAM-1 resulting in reduced release of proinflammatory mediators such as IL-4 and IgE leading to the prevention of inflammation. MMP-9 is believed to play an important role in airway remodeling in chronic airway diseases and MMP-9 expression is closely related to the severity of asthma [48, 49]. As there is an inverse relationship between MMP-9 and airway hyperresponsiveness [50],* Cordyceps sinensis *may reduce leukocyte extravasation and lymphocyte accumulation in the walls of asthmatic airways by the decrease of MMP-9 expression. The precise pathways of the abovementioned effects in determining the production of cytokines remain to be further studied. Our results were in agreement with previous studies highlighting the suppressive effect of Cordyceps in the production of inflammatory mediators (IL-1, IL-6, IL-8, IL-10, TNF-*α*, iNOS) from macrophages and mast cells as well as from LPS-stimulated BALF cell cultures [51, 52].

One of the major limitations of the present study was the limited number of patients with asthma recruited from a single geographical location. Hence, a multicentric study is required to confirm the current study results. Moreover, the use of a placebo control group and the blinding to the treatment are essential methodological study designs that were not considered. Finally, the efficacy of* Cordyceps sinensis *in patients with asthma has to be evaluated at a longer duration of treatment.

In conclusion, the current study provides important novel information regarding the benefits of adding* Cordyceps sinensis *to the treatment regimen of patients suffering from asthma.* Cordyceps sinensis *improved the quality of life of the patients by reducing asthma-related symptoms and asthma onset frequency and severity, while it significantly attenuated asthma-induced inflammation at the 3-month intervention period, as demonstrated by decreased expression of inflammatory biomarkers. These novel findings provide useful information of the use of* Cordyceps sinensis* in the asthma treatment in human populations. In summary,* Cordyceps sinensis *is an effective Chinese medicine for asthma patients without additional safety risks.

## Figures and Tables

**Table 1:** Characteristics of the 120 subjects at baseline assessment.

	Total	Control group (*n* = 60)	Treatment group (*n* = 60)
*Demographic characteristics*			
Gender: male/female	54/66	40/20	34/26
Age (years): medium (IQR)	53.50 (46, 63)	56 (50, 63)	52 (45.25, 61)
Duration of asthma (years): medium (IQR)	10 (5, 19.5)	7.5 (4, 12)	10.5 (8, 20)
Current smoker: *n* (%)	34 (28.3)	16 (26.7)	18 (30.0)
BMI	24.14 (3.65)	24.13 (3.49)	24.15 (3.84)
*Asthma medications*			
Inhaled corticosteroids (ug/d): medium (IQR)	500 (500, 700)	500 ± (500, 750)	500 ± (200, 750)
Oral steroids (maintenance): *n* (%)	20 (16.6)	9 (15.0)	11 (18.3)
Long-acting *β*_2_ agonists: *n* (%)	15 (12.5)	8 (13.3)	7 (11.7)
Anticholinergics: *n* (%)	25 (20.8)	14 (23.3)	11 (18.3)
Theophylline: *n* (%)	11 (9.16)	5 (8.33)	6 (10.0)
Leukotriene antagonists: *n* (%)	15 (12.5)	7 (11.7)	8 (13.3)
*Clinical symptoms*			
Daytime exacerbation: medium (IQR)	5 (2, 10)	5 (4, 6)	7 (1, 12)
Nighttime exacerbation: medium (IQR)	6 (2, 10)	10 (5, 10)	4 (2, 9)
Nighttime awakenings: medium (IQR)	12 (5, 20)	15 (10, 20)	11 (5, 18.75)
Asthma symptom-free days: medium (IQR)	25 (10, 45)	15 (0, 40)	25.5 (10, 52.5)
Rescue-free days: medium (IQR)	0 (0, 38.75)	10 (0, 45)	2 (0, 27.5)

Daytime exacerbation: daytime exacerbations during the 90 days that preceded visit.

Nighttime exacerbation: nighttime exacerbations during the 90 days that preceded visit.

Nighttime awakenings: nighttime awakenings during the 90 days that preceded visit.

Symptom-free days: days free of asthma symptoms during the 90 days that preceded visit.

Rescue-free days: days free of rescue medication during the 90 days that preceded visit.

IQR: interquartile range between upper quartile and lower quartile.

**Table 2:** AQLQ scores at baseline, immediately following the intervention period, and at the 3-month follow-up assessment.

	Control group (*n* = 60)			Treatment group (*n* = 60)			*z*	*p*
	Baseline	Postintervention	3-month follow-up	Baseline	Postintervention	3-month follow-up		
*AQLQ*								
Activity limitation	5.46 (4.92, 5.92)	5.58 (4.33, 5.92)	5.25 (4.00, 5.67)	5.08 (4.50, 5.67)	5.25 (4.33, 6.17)	5.50 (4.33, 6.17)^ac^	2.446	0.014
Asthma symptom	5.78 (4.78, 6.67)	5.89 (5.00, 6.44)	6.05 (5.56, 6.33)^b^	5.61 (4.89, 6.33)	6.11 (5.22, 6.67)^a^	6.27 (5.33, 6.78)^ac^	2.382	0.037
Emotional function	5.50 (5.20, 6.40)	6.20 (3.60, 6.40)	5.80 (5.00, 6.44)	5.40 (4.60, 6.20)	5.70 (4.80, 6.60)	6.10 (5.20, 6.60)^bd^	2.667	0.008
Environment stimuli	6.60 (5.60, 7.00)	6.40 (5.60, 6.80)	6.00 (5.00, 6.20)	6.20 (5.40, 7.00)	6.20 (5.20, 7.00)	6.20 (5.00, 7.00)	1.219	0.223
Overall average	5.63 (5.20, 5.91)	5.80 (4.94, 6.06)	5.60 (4.57, 6.20)	5.44 (3.25, 5.50)	5.78 (5.14, 6.51)^a^	5.81 (4.80, 6.20)^bc^	2.244	0.049

Data are medium (IQR). ^a^ *p* < 0.05 and ^b^ *p* < 0.01 versus baseline; ^c^ *p* < 0.05 and ^d^ *p* < 0.01 CS group versus control group.

**Table 3:** Lung function scores and serum cytokines levels at baseline and immediately following the intervention period.

	Control group (*n* = 60)		Treatment group (*n* = 60)		*z*	*p*
	Baseline	Postintervention	Baseline	Postintervention		
*Lung function*						
FEV1 predicted, %	56.50 (33.40, 63.62)	60.50 (36.25, 66.87)	58.50 (40.75, 68.00)	63.10 (43.70, 72.60)^c^	2.442	0.015
PEFR, %	48.05 (33.45, 71.27)	50.50 (34.55, 64.85)	58.30 (36.62, 65.65)	59.09 (44.82, 67.70)^c^	2.525	0.012
FEV1/FVC predicted, %	57.54 (47.74, 71.29)	56.68 (45.96, 75.33)	54.01 (44.22, 63.00)	57.36 (49.77, 67.67)^bc^	2.162	0.031
*Serum biomarkers*						
IgG (mg/mL)	58.59 (31.45, 85.93)	61.77 (30.59, 84.78)	40.53 (24.99, 50.78)	45.74 (38.43, 57.45)^bc^	2.459	0.014
IgE (ng/mL)	19.37 (15.75, 30.51)	24.82 (19.84, 47.65)	31.22 (16.44, 51.27)	26.21 (22.63, 31.90)^bd^	3.655	0.000
ICAM-1 (ng/mL)	266.43 (209.99, 314.51)	324.34 (257.65, 400.17)	313.12 (255.55, 363.66)	291.35 (233.08, 311.71)^ad^	4.462	0.000
IL-4 (pg/mL)	60.67 (32.92, 79.50)	63.00 (35.75, 75.15)	65.60 (46.67, 77.00)	47.33 (30.67, 64.67)^bc^	2.529	0.011
IFN-*γ* (pg/mL)	8.45 (7.25, 9.15)	8.40 (5.85, 9.35)	9.00 (6.20, 12.00)	8.50 (7.20, 11.80)	1.126	0.260
MMP-9 (ng/mL)	537.50 (256.43, 626.34)	644.64 (379.24, 626.34)	719.64 (504.46, 1238.88)	410.63 (272.62, 657.14)^bd^	5.092	0.000

Data are medium (IQR). ^a^ *p* < 0.05 and ^b^ *p* < 0.01 Postinterventions. Baseline; ^c^ *p* < 0.05 and ^d^ *p* < 0.01 CS group versus control group.

FEV1 (%): forced expiratory volume in one second (percentage of predicted).

PEF (%): mean peak expiratory flow (% predicted).

FVC: forced vital capacity.

**Table 4:** Asthma onset infrequency, severity, and medication change at baseline and immediately following the intervention period.

	Control group (*n* = 60)		Treatment group (*n* = 60)		*z*	*p*
	Baseline	Postintervention	Baseline	Postintervention		
Daytime onset	5 (4, 6)	5.5 (4, 10)	7 (1, 12)	2 (0, 5)^bc^	2.589	0.010
Nighttime onset	10 (5, 10)	7 (2, 8)	4 (2, 9)	2 (0, 5)^b^	1.831	0.067
Nocturnal awakenings	15 (10, 20)	15 (10, 15)	11 (5, 18.75)	5.5 (3, 13.75)^b^	1.355	1.175
Symptom-free days	15 (0, 40)	20 (12, 45)	25.5 (10, 52.5)	47.50 (22.75, 57.25)^bd^	4.072	0.000
Rescue-free days	10 (0, 45)	20 (0, 30)	8 (0, 37.5)	32.50 (12.50, 49.00)^bd^	4.679	0.000
Average inhaled corticosteroids (ug/d)	500 (500, 750)	550 (500, 800)	500 (500, 687.5)	500 ± (200, 750)^b^	1.610	0.108

Data are medium (IQR), ^a^ *p* < 0.05 and ^b^ *p* < 0.01 versus baseline. ^c^ *p* < 0.05 and ^d^ *p* < 0.01 CS group versus control group.

**Table 5:** Self-reported symptoms during treatment of study.

	Control group (*n* = 60)	Treatment group (*n* = 60)
Headache	1 (1.6%)	1 (1.6%)

Dizziness	0 (0.0%)	2 (3.3%)
Throat discomfort	2 (3.3%)	2 (3.3%)
Dry mouth	3 (5.0%)	2 (3.3%)
Diarrhea	0 (0.0%)	1 (1.6%)
